# Platelet-Rich Plasma (PRP) bidirectionally modulates scar formation via the Piezo1-YAP/TAZ axis: a novel mechanotransduction hypothesis

**DOI:** 10.3389/fcell.2026.1734266

**Published:** 2026-01-26

**Authors:** Ju Tian, Jieyu Yang, Wenjie Fu, Biao Cheng

**Affiliations:** 1 Department of Plastic Surgery, Zhongshan City People’s Hospital, Zhongshan, Guangdong, China; 2 Department of Plastic Surgery, Zhongshan City People’s Hospital Affiliated to Shenzhen University, Zhongshan, Guangdong, China; 3 Department of Plastic Surgery, General Hospital of Southern Theater Command, People’s Liberation Army, Guangzhou, Guangdong, China

**Keywords:** fibroblast, mechanotransduction, Piezo1 - YAP/TAZ, Platelet - Rich Plasma (PRP), scar formation

## 1 Introduction

Platelet-Rich Plasma (PRP), a core biotherapeutic material in regenerative medicine, exhibits unique advantages in wound healing and tissue regeneration owing to its high concentrations of platelets and diverse bioactive factors, including albumin, fibrinogen, globulins, and growth factors such as Transforming growth factor-β1 (TGF-β1), Platelet-derived growth factor (PDGF), and Vascular endothelial growth factor (VEGF) (Everts et al., 2024). However, the paradoxical bidirectional regulatory effects observed in its clinical application have garnered increasing attention: while PRP accelerates wound healing by promoting cell proliferation, angiogenesis, and ordered collagen deposition, it may also induce hypertrophic scars or even keloids due to excessive fibroblast activation and dysregulated extracellular matrix (ECM) metabolism (Chen et al., 2018; Ebrahimi et al., 2022). This bidirectional effect underscores an urgent unmet clinical need: the lack of mechanistic understanding of PRP’s scar-modulating pathways has prevented the development of precision strategies to maximize its therapeutic potential while mitigating fibrotic risks. Current research remains limited to broad biochemical signaling or generic ECM remodeling (Yang and Zhang, 2025; Wu et al., 2025), with no focus on the mechanotransduction networks that bridge PRP’s biological and physical effects on scar tissue.

Recent advances in mechanotransduction research have shown that cellular sensing and response to matrix mechanical cues (e.g., stiffness, tension) are critical regulators of ECM metabolism and scar fate. Among these, Piezo1—the first identified mechanosensitive cation channel in eukaryotes (non-selective, permeable to Na^+^, Ca^2+^, and other ions) (Fang et al., 2021; Coste et al., 2010)—is overexpressed in scar tissue, responds to mechanical stretch, and drives fibroblast activation and hypertrophic scar formation (He et al., 2021; Rennekampff et al., 2024). The interplay between its activation and YAP/TAZ nuclear translocation serves as the central mechanism by which mechanical and certain chemical signals influence scar outcomes (Hasegawa et al., 2021; Liu et al., 2021; Rashidi et al., 2025). In skin fibrosis, Piezo1 forms a positive feedback loop by sensing tissue stiffness: its upregulation promotes a fibroblast proliferative phenotype, whereas Piezo1-knockout fibroblasts lose their fibrotic traits even on high-stiffness substrates (He et al., 2024; Li et al., 2022). Piezo1-YAP/TAZ is consistently implicated in scar pathogenesis: Piezo1 is overexpressed in hypertrophic scar/keloid fibroblasts and YAP/TAZ nuclear translocation directly promotes fibroblast activation and ECM deposition (He et al., 2021; Rennekampff et al., 2024; Rashidi et al., 2025). No other pathway has been shown to integrate PRP’s dual biochemical-mechanical actions, making it the most relevant and translatable target for optimizing PRP therapy. Beyond skin, Piezo1 has been implicated in pulmonary fibrosis (Xu et al., 2025), cardiac fibrosis (Jin et al., 2024), and liver fibrosis (Wang et al., 2024), suggesting its broader role in mechanotransduction-driven fibrotic diseases. However, its mechanistic contribution may vary across organs and contexts.

Previous studies have suggested that PRP may activate or synergize with the Piezo1 channel via its abundant growth factors and potential lipid components, concurrently triggering critical Ca^2+^ signaling cascades that drive angiogenesis, cell proliferation, differentiation, and matrix remodeling—ultimately promoting the regeneration of damaged myocardial tissue (Alharbi et al., 2022). Regarding whether PRP can directly activate the Piezo1 channel, current evidence lacks direct experimental validation; however, indirect correlations and theoretical support have been proposed: the platelet plasma membrane in PRP may supply phosphatidylinositol bisphosphate (PIP_2_), a lipid molecule recognized as a co-factor for Piezo1 channel activation (Alharbi et al., 2022; Smith et al., 2025; Yang et al., 2004; Borbiro et al., 2015-02).

Despite the well-established pro-regenerative effects of PRP, a critical knowledge gap persists in understanding whether PRP modulates fibroblast behavior and scar progression through the Piezo1-mediated mechanotransduction pathway. Based on our understanding of PRP’s active components, Piezo1’s functional roles (mechanosensing and pro-fibrotic activity), and their established regulatory crosstalk, we propose the following plausible hypothesis: PRP bidirectionally regulates scar formation by activating the Piezo1 channel on fibroblast surfaces, thereby modulating fibroblast proliferation, differentiation, and ECM metabolism. This framework not only clarifies PRP’s molecular mechanism of action but also provides a targeted strategy to optimize clinical outcomes (e.g., Piezo1 inhibitors, injection parameter tuning), filling the unmet need for precision anti-scar therapies.

## 2 Hypothesis

**FIGURE 1:**
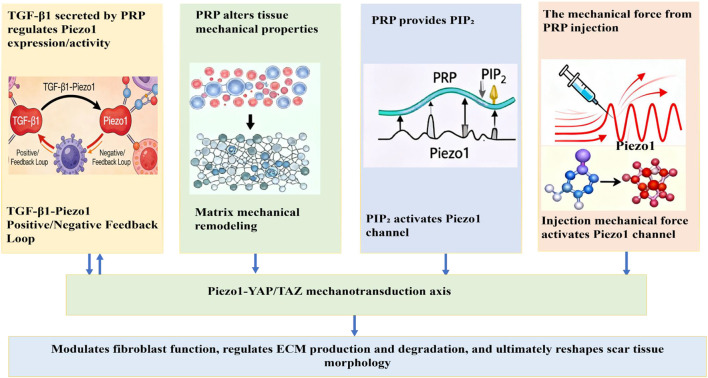
PRP bidirectionally modulates fibroblast function and scar progression by targeting the Piezo1-YAP/TAZ mechanotransduction axis via multidimensional biological effects.

We propose that PRP bidirectionally modulates fibroblast function and scar progression by targeting the Piezo1-YAP/TAZ mechanotransduction axis via multidimensional biological effects (Figure 1). During physiological repair, this modulation attenuates scar formation; during pathological fibrosis, it promotes the development of hypertrophic scars or keloids. We further hypothesize that PRP regulates the Piezo1 channel through the following mechanisms:

1. TGF-β1 secreted by PRP upregulates Piezo1 expression/activity, and the two interact via positive/negative feedback loops to modulate scar formation (Zhao et al., 2022; Holt et al., 2025; Yang et al., 2025; Cai et al., 2023).
2. PRP alters tissue mechanical properties (e.g., stiffness) by inducing cell proliferation and matrix remodeling. Changes in the stiffness of its contained fibrin matrix further tune the tension of the tissue mechanical microenvironment, thereby regulating Piezo1 channel activity.
3. The platelet plasma membrane in PRP may supply phosphatidylinositol bisphosphate (PIP_2_)—a lipid molecule known to activate the Piezo1 channel (Alharbi et al., 2022; Smith et al., 2025; Yang et al., 2004; Borbiro et al., 2015-02).
4. During PRP injection therapy, mechanical force from the injection directly activates the Piezo1 channel. PRP injection may also activate mechanosensitive Piezo1 channels by locally increasing tissue tension.

## 3 Evolution of the hypothesis

The core pathological process of hypertrophic scarring (e.g., in skin wounds and visceral organs like the lung or liver) involves excessive fibroblast activation driven by dysregulated mechanotransduction pathways (Griffin et al., 2021) and aberrant ECM deposition orchestrated by profibrotic signaling hubs such as YAP/TAZ in pulmonary fibrosis (Papavassiliou et al., 2024). As a mechanosensitive ion channel, Piezo1 primarily regulates cellular functions (e.g., fibroblast proliferation, collagen secretion) by sensing extracellular mechanical cues (e.g., tissue tension, matrix stiffnes) or indirectly via growth factor signaling (He et al., 2024; Li et al., 2022). These functions exhibit bidirectional roles in physiological repair and pathological fibrosis—findings that strongly support the feasibility of targeting Piezo1 for scar therapy. Piezo1 activation in physiological tissue repair occurs through physiological mechanical cues​ (e.g., wound-edge contraction, collagen matrix remodeling) and regulated growth factor signaling. Moderate Piezo1 activation, as part of fibroblast functional regulation, facilitates normal tissue healing and reduces scar overgrowth (Rashidi et al., 2025; Emig et al., 2021). In contrast, pathological fibrosis arises from a synergistic interplay between Piezo1 hyperactivation (triggered by persistent mechanical stretch or excess profibrotic factors) and other pathological elements (e.g., chronic inflammation, aberrant ECM deposition). Activated Piezo1 further amplifies TGF-β1-downstream profibrotic signals via calcium signaling (forming a positive feedback loop) and activates pathways like YAP/TAZ. This drives uncontrolled fibroblast proliferation and excessive ECM deposition, potentially promoting hypertrophic scar or keloid formation (He et al., 2024; Bartoli et al., 2022).

The biological effects of PRP depend primarily on bioactive molecules released upon platelet activation: beyond growth factors, these include the fibrin scaffold formed post-activation. This scaffold not only provides physical support for cell migration/adhesion but also modulates cell behavior via mechanical properties (e.g., matrix stiffness) (Zhou et al., 2023). Based on current mechanistic insights, PRP bidirectionally regulates fibroblast function and scar progression by targeting the Piezo1-YAP/TAZ mechanotransduction axis via multidimensional effects. Below, we elucidate the underlying mechanisms:

### 3.1 PRP regulates scar progression via a TGF-β1-Piezo1 positive/negative feedback loop

As a platelet-enriched reservoir of bioactive factors, PRP’s key effector—TGF-β1 is initially present as a latent complex (latent TGF-β1, L-TGF-β1) embedded in the ECM (Klingberg et al., 2014). In fibrotic contexts, fibroblast contractions (amplified by matrix stiffness and Piezo1-mediated mechanosignaling) drive ECM remodeling to release and activate L-TGF-β1 (Swain et al., 2022). Activated TGF-β1 then regulates Piezo1 mRNA transcription and membrane protein expression via the classical Smad-dependent pathway (Smad2/3 phosphorylation) and non-classical MAPK/YAP pathway (Liu et al., 2021; Holt et al., 2025; Chen et al., 2025; Andolfo et al., 2020). Notably, Piezo1 activation mediates calcium (Ca^2+^) influx, which, via calmodulin-dependent kinase II (CaMKII), phosphorylates TGF-β1 receptor I (TβRI). This enhances downstream TGF-β1-Smad signaling, forming a “TGF-β1↑→Piezo1↑→amplified TGF-β1 signaling” positive feedback loop (He et al., 2024; Holt et al., 2025). During physiological repair, this loop maintains ECM homeostasis (synthesis vs. degradation) and limits scar formation. However, in pathological fibrosis, the loop is markedly amplified: In hypertrophic scars, elevated matrix stiffness exacerbates fibrosis via the Piezo1-Wnt2/Wnt11-CCL24 inflammatory pathway—while increased stiffness further upregulates Piezo1, creating a “stiffness↑→Piezo1↑→fibrosis↑” vicious cycle (He et al., 2024). Mechanically activated Piezo1-YAP signaling upregulates factors like CCN1/CCN2, driving fibroblast proliferation and collagen deposition to strengthen pro-fibrotic loops.

A similar mechanism operates in pulmonary fibrosis, where TGF-β1/Smad synergizes with molecules like SULF1/NEU3 to promote ECM deposition (Xu et al., 2025; Tu et al., 2024). For targeted intervention, inhibiting Piezo1 (e.g., with the inhibitor OB-1) blocks TGF-β1 signaling, reduces aberrant collagen deposition, and disrupts the fibrotic loop (Holt et al., 2025). Conversely, moderate Piezo1 activation during physiological repair is critical for cell migration and angiogenesis—its depletion causes epithelial barrier dysfunction (Holt et al., 2021).

In summary, the Piezo1-TGF-β1 positive feedback loop is a core node in fibrosis progression. Its dynamic balance dictates whether tissue repair adopts a physiological (scar-minimizing) or pathological (fibrotic/hypertrophic) trajectory. This framework not only explains PRP’s dual effects on scar formation but also identifies Piezo1 as a precision target for optimizing PRP-based therapies—e.g., balancing its pro-regenerative and pro-fibrotic activities via mechanistic modulation.

### 3.2 PRP indirectly regulates Piezo1 activity via matrix mechanical remodeling

The fibrin matrix in PRP modulates porosity and elastic modulus, which may influence cell migration and angiogenesis (He et al., 2024; Li et al., 2022). For instance, combining PRP with microvascular fragments (MVF) accelerates wound healing and markedly boosts neovascular density (Laschke and Menger, 2022; Dinter et al., 2024). In this therapeutic context, MVF synergize with PRP to enhance neovascularization in chronic wounds by integrating into existing vasculature and stimulating capillary formation, while also improving tissue perfusion and nutrient delivery (Dinter et al., 2024). Together, these actions foster an optimal microenvironment that reduces scar formation. PRP-induced fibroblast proliferation and ECM remodeling significantly alter the local mechanical microenvironment of tissues. On one hand, PRP promotes fibroblast secretion of ECM components (e.g., collagen, fibronectin) to increase substrate stiffness; on the other hand, PRP inhibits ECM deposition by regulating fibroblast function (Cao et al., 2022). Additionally, the fibrin matrix in PRP—by modulating porosity and elastic modulus—may influence cell migration and angiogenesis (Gruber, 2000; Pauty et al., 2021). For example, PRP combined with MVF accelerates wound healing and boosts neovascular density (Dinter et al., 2024). As a mechanosensitive ion channel, the open probability of Piezo1 highly depends on ECM physical properties: elevated matrix stiffness triggers Piezo1 conformational rearrangement, enhancing interactions between its intracellular domains and ankyrin/vimentin. This promotes Ca^2+^ influx and activates downstream YAP/TAZ signaling, driving scar formation (Yan et al., 2023). Conversely, during physiological repair, moderate matrix remodeling induced by PRP limits Piezo1 overactivation, suppresses YAP/TAZ nuclear translocation, and maintains fibroblast quiescence while facilitating ECM degradation (Rashidi et al., 2025). Notably, YAP/TAZ nuclear localization correlates positively with stiffness in 2D cultures but may show inverse/nonlinear relationships in 3D environments (Scott et al., 2021); its regulation is context-dependent (e.g., dimensionality, cell type) and involves crosstalk with pathways (e.g., Hippo, Wnt). Targeting this axis (e.g., Piezo1 genetic knockdown/knockout (Holt et al., 2021; Mei et al., 2024), GsMTx4-mediated Piezo1 inhibition (Liao et al., 2025), or YAP inhibitors) holds therapeutic promise for fibrosis and cancer, though tissue specificity remains essential to preserve physiological repair.

### 3.3 Mechanical forces from PRP injection directly activate Piezo1 channels

Piezo1 expression is significantly higher in fibroblasts from pathological scars (e.g., hypertrophic scars, keloids) versus normal skin, and it positively correlates with YAP signaling. The Piezo1-YAP pathway is effectively inhibited by the Piezo1 inhibitor GsMTx4 (Liao et al., 2025). Piezo1 responds to mechanical forces in a threshold-dependent manner (typically requiring >25 mN/m membrane tension) (Ridone et al., 2019). During clinical PRP application, multiple injections (2–5 injections per standard therapy cycle, at 1–2 weeks intervals) are applied, and the transient mechanical forces (shear stress, pressure gradients, and membrane stretch) generated by each syringe injection are critical to trigger Piezo1 channel opening—with repeated injections further potentiating such Piezo1 activation. The shear stress and pressure induced by PRP injection are likely comparable to the natural physiological shear stress and mechanical strain that drive endogenous Piezo1-mediated fibroblast activation *in vivo* (Lai et al., 2022), though this has not been experimentally validated in existing studies. Such clinically relevant mechanical stimulation thus triggers Piezo1 channel gating on fibroblasts, and repetitive injection amplifies this mechanotransduction cascade to regulate fibroblast proliferation and differentiation, mirroring the physiological dynamics of Piezo1 activation *in vivo* (Lai et al., 2022).

In summary, PRP’s regulation of scar progression fundamentally integrates multidimensional signals: biochemical factors, matrix mechanics, and mechanical forces. Specifically: TGF-β1 modulates basal Piezo1 expression via transcriptional/post-translational modifications; Matrix remodeling indirectly regulates Piezo1 activity by altering mechanical properties; Injection mechanical forces directly activate Piezo1 channels. These components synergistically act on the Piezo1-YAP/TAZ axis: they maintain ECM metabolic homeostasis (inhibiting scar hyperplasia) during physiological repair and disrupt this balance (promoting keloid/hypertrophic scar formation) during pathological fibrosis. This mechanistic framework provides a theoretical basis for the precision application of PRP in scar therapy—such as modulating PRP concentration and delivery methods (topical/injection) to optimize mechanical force stimulation.

## 4 Implications of the hypothesis

The hypothesis that PRP bidirectionally modulates scar formation by targeting the Piezo1-YAP/TAZ mechanotransduction axis via multidimensional biological effects reveals the synergism of biochemical factors, tissue mechanics, and cellular mechanotransduction in wound repair—offering critical insights for both basic research and clinical translation:

### 4.1 Elucidating biochemical-mechanical signal crosstalk in scar pathogenesis

TGF-β1 in PRP not only directly drives fibroblast proliferation and collagen deposition but also upregulates Piezo1 expression via Smad2/3-mediated transcriptional activation: phosphorylated Smad2/3 binds to the *Piezo1* gene promoter to induce its transcription (Andolfo et al., 2020). Activated Piezo1, in turn, amplifies TGF-β1 downstream signaling through calcium influx, forming a positive feedback loop: TGF-β1↑ → Piezo1↑ → amplified TGF-β1 signaling. This mechanism explains the self-reinforcing cycle of “excessive ECM deposition-fibrosis progression” in pathological scars (e.g., hypertrophic scars) due to uncontrolled positive feedback. It also clarifies the pivotal role of moderate feedback activation (maintaining ECM homeostasis) in physiological repair—providing a novel perspective for understanding organ fibrosis (e.g., lung, liver).

### 4.2 Proposing tissue mechanical microenvironment as a novel target for scar regulation

PRP-mediated regulation of Piezo1 activity via matrix mechanical remodeling transcends the traditional paradigm of molecule-only interventions for scar modulation. This highlights that the anti-fibrotic and pro-regenerative effects of PRP on scar tissue arise not only from its constituent growth factors (e.g., TGF-β1, PDGF) but also from its ability to remodel the ECM—including driving collagen cross-linking and fibrin network assembly—to dynamically alter tissue biomechanical stiffness. Such matrix stiffness changes directly modulate the open probability of Piezo1 mechanosensitive channels in resident fibroblasts: pathologically stiff scar matrices excessively activate the Piezo1-YAP/TAZ mechanotransduction axis to promote persistent fibrosis and pathological scar formation, whereas physiologically moderate matrix stiffness restricts Piezo1 overactivation and maintains fibroblast phenotypic homeostasis. Collectively, these findings indicate that future scar therapeutic strategies could be optimized by combining PRP with matrix-softening agents [e.g., lysyl oxidase (LOX) inhibitors (Yu et al., 2025)] or by fine-tuning PRP-induced matrix remodeling (e.g., via controlled fibrin concentration) to normalize tissue stiffness and Piezo1 activation, thereby maximizing therapeutic efficacy while mitigating excessive fibrosis.

### 4.3 Guiding optimization of mechanical parameters for clinical PRP application

The direct activation of Piezo1 by mechanical forces from PRP injection links clinical procedures (e.g., injection force, frequency) to underlying mechanisms. Transient mechanical forces during injection—such as shear stress and pressure gradients—can directly trigger Piezo1 channel opening. For example,: High-concentration/rapid injection may cause excessive Piezo1-YAP/TAZ activation (exacerbating fibrosis) due to overstimulation (Jiang et al., 2025); Low-dose/slow injection avoids excessive mechanical cues (promoting physiological repair).

This provides a rationale for optimizing clinical PRP protocols: injection parameters (e.g., needle gauge, single-dose volume) should be tailored to scar type (hypertrophic/atrophic). Additionally, mechanical modulation techniques (e.g., combined ultrasound/acoustic radiation force) can precisely regulate Piezo1 activity.

Based on this hypothesis, future scar treatment must shift from a “one-size-fits-all” approach to a personalized, mechanism-driven model: Stratify patients into “high fibrosis risk” or “delayed healing” subgroups using biomarkers (e.g., Piezo1 expression, matrix stiffness, TGF-β1 levels); Tailor PRP concentration, injection frequency, or combination therapies (e.g., with anti-fibrotic agents) to subgroup needs; Monitor changes in the mechanical microenvironment during treatment (via advanced imaging/sensors) to dynamically optimize protocols.

In summary, this hypothesis not only deepens the mechanistic understanding of PRP-mediated scar modulation, with Piezo1 identified as a key mechanosensitive effector (He et al., 2023) and potential therapeutic target for anti-fibrotic scar therapy, but also provides a robust theoretical framework for developing next-generation scar therapies that integrate biochemical, mechanical and clinical parameters.

## 5 Testing the hypothesis

To systematically validate the hypothesis, a hierarchical experimental strategy can be designed, integrating *in vitro* mechanistic dissection, *in vivo* model testing, and multi-pathway synergistic blockade—centered on three core mechanisms: the TGF-β1-Piezo1 positive feedback loop, matrix mechanical regulation, and direct activation by PRP injection forces (Table 1).

**TABLE 1:** Experimental and theoretical validation strategies.

Validation objective	Experimental approach	Key methods and techniques	Expected outcomes	Validation objective
Validate the TGF-β1-Piezo1 positive feedback loop	*In vitro*: Cultured human skin fibroblasts (HSFs) stimulated with PRP (containing endogenous TGF-β1). *In vivo*: Mouse scar model (PRP-treated vs. PRP + TGF-β1 neutralizing antibody)	- Quantify TGF-β1 concentration (ELISA). - Analyze Piezo1 mRNA/protein expression (qPCR, Western blotting). - Calcium imaging to monitor Piezo1 activation. - Assess Smad2/3 phosphorylation (TGF-β signaling downstream). - Immunofluorescence for Piezo1/TGF-β1 colocalization (*in vivo*)	- Correlation between TGF-β1 levels and Piezo1 expression. - Reduced Piezo1 expression upon TGF-β1 neutralization. - Blocked fibrosis markers (e.g., collagen deposition) in antibody-treated groups. - Confirmed feedback loop drives pathological scar progression	Validate the TGF-β1-Piezo1 positive feedback loop
Evaluate matrix mechanical regulation of Piezo1	*In vitro*: HSFs cultured on collagen gels with stiffness gradients (mimicking physiological/pathological ECM). Co-cultured with PRP to simulate matrix remodeling. *In vivo*: PRP-treated mouse scars; assess tissue stiffness (AFM) and fibrosis markers	- Measure matrix stiffness (AFM). - Track Piezo1 activity (calcium imaging). - Detect YAP nuclear translocation (Western blotting). - Treat cells with LOX inhibitor (reduces stiffness) to isolate mechanical effects. - Histological analysis (Masson’s trichrome, α-SMA) *in vivo*	- Stiff matrices upregulate Piezo1/YAP signaling and fibrosis markers. - Physiological stiffness limits Piezo1 overactivation. - YAP nuclear localization correlates with stiffness. - Matrix softening (e.g., LOX inhibition) reduces fibrosis via Piezo1 suppression	Evaluate matrix mechanical regulation of Piezo1
Test direct activation of Piezo1 by injection forces	*In vitro*: Simulate PRP injection using a pressure pump + nanopipette tension system; apply controlled shear stress/pressure to cells *In vivo*: Compare low- vs. high-rate PRP injections in mouse scars; assess mechanical force effects	- Observe Piezo1-GFP fluorescence (channel opening). - Calcium imaging for Ca^2+^ influx. - Use Gd^3+^ (mechanosensor blocker) to confirm Piezo1 specificity. - Quantify Piezo1 membrane levels (WB) and collagen deposition (IHC) *in vivo*.- Monitor force-induced YAP/TAZ activation	- High mechanical forces (e.g., rapid injection) trigger Piezo1 opening and fibrosis-related signals. - Low-force injection minimizes Piezo1 activation, supporting physiological repair. - Force rate correlates positively with fibrosis severity. - Confirmed mechanical force directly regulates Piezo1 activity	Test direct activation of Piezo1 by injection forces

For the TGF-β1-Piezo1 positive feedback loop, *in vitro* studies can use human skin fibroblasts (HSFs) stimulated with PRP (containing endogenous TGF-β1). TGF-β1 concentration changes can first be quantified via ELISA, while Piezo1 mRNA/protein expression can be analyzed using qPCR and Western blotting (WB) to explore their correlation. To confirm the TGF-β1→Piezo1 axis, TGF-β1 can be neutralized with antibodies or its receptor I (TβRI) can be knocked down via siRNA; reduced Piezo1 expression can validate upstream control. Calcium imaging can monitor Ca^2+^ influx upon Piezo1 activation, while Smad2/3 phosphorylation assays can indirectly link Piezo1 function to TGF-β signaling (Zhao et al., 2022; Yang et al., 2025). *In vivo*, a mouse scar model can be generated, with groups receiving PRP alone or PRP plus TGF-β1 neutralizing antibody. Immunofluorescence co-localization can assess Piezo1/TGF-β1 subcellular distribution, and WB for YAP nuclear translocation (a mechanotransduction hallmark) can test whether this feedback drives fibrosis (Rashidi et al., 2025; Liao et al., 2025; Xue et al., 2025; Peng et al., 2025).

To evaluate matrix mechanical regulation, *in vitro* experiments can use collagen gels with stiffness gradients to mimic physiological/pathological microenvironments. HSFs co-cultured with PRP on these gels can be analyzed via atomic force microscopy (AFM) to quantify local matrix stiffness changes in the pericellular microenvironment surrounding individual fibroblasts (rather than bulk/overall stiffness of the gel), as AFM enables high-resolution nanomechanical mapping of pericellular matrix stiffness with spatial precision at the single-cell scale (Shioka et al., 2024; Adam et al., 2025). Calcium imaging will be employed to monitor Piezo1 channel activity in response to these localized mechanical cues (Xue et al., 2025; Peng et al., 2025), and Western blotting (WB) to assess nuclear translocation of YAP (a downstream effector of Piezo1 mechanotransduction) (Rashidi et al., 2025).

To test if matrix stiffness acts via Piezo1-YAP, cells can be treated with a lysyl oxidase (LOX) inhibitor (reducing stiffness by blocking collagen cross-linking) (Yu et al., 2025), and changes in Piezo1 activity and YAP translocation can be compared. *In vivo*, PRP-treated mouse scars can be assessed post-surgery: AFM can measure tissue stiffness, immunohistochemistry (IHC) can quantify collagen density (Masson’s trichrome) and α-SMA (fibroblast activation) to grade fibrosis (Lin et al., 2025), and immunofluorescence double labeling can evaluate Piezo1/YAP co-localization (Zhuang et al., 2024). These studies can elucidate how Piezo1 senses matrix stiffness to activate YAP signaling, driving fibrosis.

For direct activation by injection mechanical force, *in vitro* experiments can simulate PRP injection using a pressure pump, combined with nanopipette tension manipulation (Lüchtefeld et al., 2024) and porous cell stretching systems (Zhu et al., 2025) for precise force control. Piezo1-GFP conformational fluorescence imaging can be used to observe Piezo1 channel opening: the GFP fusion tag reports on Piezo1’s mechanosensitive structural rearrangements upon channel gating (this is distinct from constitutive GFP fluorescence that only indicates Piezo1 protein presence, with no distinction between closed and open states) (Mulhall et al., 2023). Calcium imaging can measure Piezo1-dependent Ca^2+^ influx, and Gd^3+^ pretreatment can confirm Piezo1 as the key mechanosensor mediating this mechanotransduction response (Malko et al., 2023). *In vivo*, low-vs. high-rate PRP injections can create mechanical gradients; WB can detect Piezo1 membrane levels, and IHC can analyze collagen deposition to link membrane tension to mechanosensing (Mills et al., 2022; Sun et al., 2022), confirming a positive correlation between force rate and fibrosis. Finally, combined *in vitro* blockade (neutralizing antibodies, LOX inhibitors, Gd^3+^) and *in vivo* combination therapies can validate that these three pathways synergize via a TGF-β1→matrix remodeling →mechanical force cascade to regulate the Piezo1-YAP axis.

Collectively, this hypothesis can establish a novel mechano-biochemical framework for PRP research—advancing beyond single-factor analyses to capture interplay between biochemical signals, matrix mechanics, and mechanical forces. Validation can optimize PRP clinical use (e.g., adjusting injection parameters, combining with Piezo1 inhibitors) and can provide a theoretical basis for precision scar therapy targeting the Piezo1-YAP axis.

## 6 Discussion

PRP is widely used in regenerative medicine for its ability to enhance tissue repair, yet its precise molecular effects on scar formation remain unclear. Recent research underscores the importance of mechanosensitive pathways—specifically the Piezo1 ion channel and the YAP/TAZ signaling axis—in wound healing and fibrosis. Within scar tissue, Piezo1 functions as a crucial amplifier of mechanical signals, working synergistically with other pathways to intensify YAP/TAZ-dependent fibrotic processes (He et al., 2021; Rennekampff et al., 2024). The bidirectional regulation hypothesis of PRP in scar progression—where it suppresses scarring during physiological repair and promotes fibrosis in pathological conditions—offers a novel lens to interpret its intricate biological effects. At its core, this mechanism hinges on targeting the Piezo1-YAP/TAZ axis via multidimensional signals (biochemical factors, matrix mechanics, and mechanical forces), with aberrant activation of this axis already recognized as a key driver of fibrosis. The Piezo1-YAP/TAZ axis is not proposed as the “sole” mechanism but as the integrative hub that unifies PRP’s multidimensional effects (biochemical, mechanical, and physical). Unlike other pathways (e.g., PI3K/Akt, Wnt) that primarily mediate isolated biochemical signals, Piezo1-YAP/TAZ uniquely transduces both soluble factors (PRP-derived TGF-β1) and mechanical cues (ECM stiffness, injection forces)—two interconnected drivers of scar formation that were previously studied in isolation (Arioka et al., 2025). However, direct associations between PRP and Piezo1 remain poorly characterized: while PRP contains profibrotic factors like TGF-β1, it also harbors anti-inflammatory (e.g., IL-1Ra) and proangiogenic (e.g., VEGF) factors that may modulate scar outcomes through distinct pathways. This functional pleiotropy likely underlies the pronounced “dose-time-scar type” dependency of PRP’s effects—explaining the controversies in clinical efficacy [e.g., some studies report improved atrophic scars (Zare et al., 2025), whereas others note exacerbated hypertrophic scars (Chen et al., 2018)].

Mechanistically, the synergistic interplay of three pathways may underpin PRP’s bidirectional regulation: the TGF-β1-Piezo1 positive feedback loop, matrix mechanical remodeling, and direct activation by injection mechanical force. In pathological scars, excessive TGF-β1 release (e.g., from high-concentration PRP or repeated injections) may drive persistent Piezo1 activation, which in turn amplifies fibroblast proliferation and collagen deposition via YAP/TAZ—creating a “fibrosis-positive feedback amplification” vicious cycle. Conversely, in physiological repair, moderate TGF-β1 levels keep this feedback tightly controlled (e.g., through TGF-β1 receptor desensitization or antagonism by antifibrotic factors), restricting Piezo1 to transient activation that maintains ECM homeostasis. Matrix mechanical remodeling, by altering tissue stiffness (e.g., increased stiffness in pathological scars from PRP-induced collagen cross-linking, or decreased stiffness in physiological repair from ordered collagen alignment), directly modulates Piezo1’s open probability: stiff matrices excessively activate the Piezo1-YAP axis to promote fibrosis, while moderate stiffness limits activation to prevent scar hyperplasia. The effect of mechanically induced Piezo1 activation also varies with injection parameters: low-dose, slow injection may trigger transient Piezo1 activation (supporting physiological repair), whereas high-dose, rapid injection delivers sustained mechanical stimulation that induces Piezo1 desensitization or dephosphorylation—exacerbating abnormal YAP/TAZ nuclear translocation (driving pathological scarring).

Among the numerous components of PRP, both TGF-β1 and PIP_2_ can directly modulate Piezo1 (Liu et al., 2021; Alharbi et al., 2022; Smith et al., 2025; Yang et al., 2004; Borbiro et al., 2015-02; Chen et al., 2025), while other components may influence certain aspects of the Piezo1-YAP/TAZ axis (Table 2). Research indicates that VEGF indirectly activates Piezo1 through a VEGF-myc-myosin 1b-Piezo1 axis (Lv et al., 2024). In this pathway, VEGF upregulates Myosin 1b expression, and the mechanical force generated by myosin contraction subsequently activates Piezo1. Furthermore, in addition to acting through the Piezo1-YAP/TAZ axis, PRP can also accelerate wound healing and re-epithelialization by releasing growth factors and exosomes that activate YAP (Guo et al., 2017; Zhang et al., 2020), which in turn influences scar formation. The YAP/TAZ axis can be activated by increased ECM stiffness and shear stresses independently of Piezo1. Conversely, in hypertrophic scars, PRP may reduce connective tissue growth factor (CTGF) via TGF-β1 negative feedback, potentially limiting excessive fibrosis (Nam and Kim, 2018). Blocking Piezo1 or modulating YAP/TAZ activity can reduce scar formation, suggesting therapeutic targets for controlling fibrosis (He et al., 2021; Rennekampff et al., 2024).

**TABLE 2:** A summary of key PRP factors and their potential effects on the Piezo1-YAP/TAZ axis.

PRP factor	Potential effect on Piezo1-YAP/TAZ axis	Mechanism
TGF-β1	Directly modulates (upregulates expression)	Activates classical Smad-dependent pathway (Smad2/3 phosphorylation) and non-classical MAPK/ERK pathway; may involve TGF-β1 negative feedback in hypertrophic scars (Liu et al., 2021; Holt et al., 2025; Chen et al., 2025; Andolfo et al., 2020)
TGF-β1-mediated feedback	Indirectly limits excessive fibrosis	Reduces CTGF in hypertrophic scars via TGF-β1 negative feedback (Nam and Kim, 2018)
PIP_2_ (lipid component)	Directly activates	Acts as a membrane co-factor for Piezo1 channel gating (Alharbi et al., 2022; Smith et al., 2025; Yang et al., 2004; Borbiro et al., 2015-02)
VEGF	Indirectly activates	Upregulates Myosin 1b expression via “VEGF-myc-myosin 1b-Piezo1 axis”; mechanical force from myosin contraction activates Piezo1 [69]
Growth factors/exosomes	No direct activation/modulation	Activates YAP to accelerate wound healing and re-epithelialization; indirectly influences scar formation (Guo et al., 2017; Zhang et al., 2020)

Despite its plausibility, the hypothesis currently lacks direct experimental validation: no studies have reported how PRP treatment affects Piezo1 expression/function in scar-associated fibroblasts, nor clarified whether PRP’s profibrotic effects depend on Piezo1. *In vivo*, the correlation between Piezo1 levels in PRP-treated scar tissue and therapeutic efficacy remains unverified. Future investigations should prioritize defining the regulatory weights of PRP components on Piezo1 and distinguishing synergistic/antagonistic interactions between profibrotic and antifibrotic factors; validating the necessity of Piezo1 in PRP-induced fibroblast activation; comparing, in animal models, the effects of different PRP preparations (concentration, activation method) combined with Piezo1 inhibitors on scar size, stiffness, and collagen deposition to establish optimal intervention parameters; and enrolling clinical cohorts to correlate pre-vs. post-treatment Piezo1 expression in scar tissue with clinical outcomes (improvement vs. worsening)—thereby translating the hypothesis into precision therapy.

## 7 Conclusion

Currently, no direct evidence confirms that PRP influences scar formation by regulating the Piezo1–YAP/TAZ axis. However, given the roles of PRP-derived TGF-β1 and Piezo1, along with their potential interaction, we hypothesize that PRP bidirectionally regulates fibroblast function and scar progression through this axis via multiple biological effects. Under physiological repair conditions, it may attenuate scarring, whereas in pathological fibrosis, it could promote hypertrophic scars or keloids. PRP might modulate Piezo1 through TGF-β1 feedback, matrix mechanical properties, PIP2 availability, and mechanical forces induced by injection. The proposed “PRP → multidimensional effects on Piezo1–YAP/TAZ → scar modulation” pathway requires experimental validation. Clarifying this mechanism is essential for understanding PRP efficacy, refining therapeutic protocols, and developing anti-scar strategies.

## References

[B1] Adam C. E. Piacenti A. R. Zhang Y. Waters S. L. Contera S. (2025). Viscoelastic time responses of polymeric cell substrates measured continuously from 0.1-5000 Hz in liquid by photothermal AFM nanorheology. Nanoscale 17 (37), 21810–21836. 10.1039/d5nr01790d 40931723

[B2] Alharbi K. S. Almalki W. H. Alzarea S. I. Kazmi I. Al-Abbasi F. A. Afzal O. (2022). A narrative review on the biology of piezo1 with platelet-rich plasma in cardiac cell regeneration. Chem-Biol Interact. 363, 110011. 10.1016/j.cbi.2022.110011 35728671

[B3] Andolfo I. Rosato B. E. Manna F. De Rosa G. Marra R. Gambale A. (2020). Gain-of-function mutations in PIEZO1 directly impair hepatic iron metabolism *via* the inhibition of the BMP/SMADs pathway. Am. J. Hematol. 95 (2), 188–197. 10.1002/ajh.25683 31737919

[B4] Arioka M. Matsunaga H. Ishikane S. Ito K. Hashimoto K. Wang Y. (2025). Celecoxib inhibits skin fibrosis *via* suppressing adipocyte progenitor-myofibroblast transdifferentiation by attenuating the YAP/TAZ signaling pathway. Life Sci. 379, 123914. 10.1016/j.lfs.2025.123914 40818661

[B5] Bartoli F. Evans E. L. Blythe N. M. Stewart L. Chuntharpursat-Bon E. Debant M. (2022). Global PIEZO1 gain-of-function mutation causes cardiac hypertrophy and fibrosis in mice. Cells 11 (7), 1199. 10.3390/cells11071199 35406763 PMC8997529

[B6] Borbiro I. Badheka D. Rohacs T. (2015). Activation of TRPV1 channels inhibits mechanosensitive piezo channel activity by depleting membrane phosphoinositides. Sci. Signal 8 (363), ra15. 10.1126/scisignal.2005667 25670203 PMC4527171

[B7] Cai G. Lu Y. Zhong W. Wang T. Li Y. Ruan X. (2023). Piezo1-mediated M2 macrophage mechanotransduction enhances bone formation through secretion and activation of transforming growth factor-β1. Cell Prolif. 56 (9), e13440. 10.1111/cpr.13440 36880296 PMC10472522

[B8] Cao Y. Li Y. Fu S. C. Shen J. Zhang H. Jiang C. (2022). Platelet-rich plasma pretreatment protects anterior cruciate ligament fibroblasts correlated with PI3K-Akt-mTOR pathway under hypoxia condition. J. Orthop. Transl. 34, 102–112. 10.1016/j.jot.2022.02.002 35891713 PMC9283994

[B9] Chen C. Wang H. Zhu G. Sun Z. Xu X. Li F. (2018). Three-dimensional poly lactic-co-glycolic acid scaffold containing autologous platelet-rich plasma supports keloid fibroblast growth and contributes to keloid formation in a nude mouse model. J. Dermatol Sci. 89 (1), 67–76. 10.1016/j.jdermsci.2017.07.020 29122407

[B10] Chen Y. Liu S. Zhou G. Sun C. Ma M. Huang R. (2025). Surficial pore structure on polyetheretherketone implants regulates mechanical properties to promote macrophage M2 polarization. ACS Appl. Mater Interfaces 17, 56861–56876. 10.1021/acsami.5c14803 41037664

[B11] Coste B. Mathur J. Schmidt M. Earley T. J. Ranade S. Petrus M. J. (2010). Piezo1 and Piezo2 are essential components of distinct mechanically activated cation channels. Science 330 (6000), 55–60. 10.1126/science.1193270 20813920 PMC3062430

[B12] Dinter M. C. Bickelmann C. Nickels R. M. Menger M. D. Laschke M. W. (2024). Microvascular fragment-loaded platelet-rich plasma dressing promotes cutaneous wound healing. Adv. Wound Care 13 (7), 336–349. 10.1089/wound.2023.0029 38299944

[B13] Ebrahimi Z. Alimohamadi Y. Janani M. Hejazi P. Kamali M. Goodarzi A. (2022). Platelet-rich plasma in the treatment of scars, to suggest or not to suggest? A systematic review and meta-analysis. J. Tissue Eng. Regen. Med. 16 (10), 875–899. 10.1002/term.3338 35795892

[B14] Emig R. Knodt W. Krussig M. J. Zgierski-Johnston C. M. Gorka O. Groß O. (2021). Piezo1 channels contribute to the regulation of human atrial fibroblast mechanical properties and matrix stiffness sensing. Cells 10 (3), 663. 10.3390/cells10030663 33809739 PMC8002259

[B15] Everts P. A. Lana J. F. Alexander R. W. Dallo I. Kon E. Ambach M. A. (2024). Profound properties of protein-rich, platelet-rich plasma matrices as novel, multi-purpose biological platforms in tissue repair, regeneration, and wound healing. Int. J. Mol. Sci. 25 (14), 7914. 10.3390/ijms25147914 39063156 PMC11277244

[B16] Fang X. Z. Zhou T. Xu J. Q. Wang Y. X. Sun M. M. He Y. J. (2021). Structure, kinetic properties and biological function of mechanosensitive piezo channels. Cell Biosci. 11 (1), 13. 10.1186/s13578-020-00522-z 33422128 PMC7796548

[B17] Griffin M. F. Borrelli M. R. Garcia J. T. Januszyk M. King M. Lerbs T. (2021). JUN promotes hypertrophic skin scarring *via* CD36 in preclinical *in vitro* and *in vivo* models. Sci. Transl. Med. 13 (609), eabb3312. 10.1126/scitranslmed.abb3312 34516825 PMC8988368

[B18] Gruber R. (2000). How to explain the beneficial effects of platelet-rich plasma. Periodontol 97 (1), 95–103. 10.1111/prd.12565 38600634 PMC11808461

[B19] Guo S. C. Tao S. C. Yin W. J. Qi X. Yuan T. Zhang C. Q. (2017). Exosomes derived from platelet-rich plasma promote the re-epithelization of chronic cutaneous wounds *via* activation of YAP in a diabetic rat model. Theranostics 7 (1), 81–96. 10.7150/thno.16803 28042318 PMC5196887

[B20] Hasegawa K. Fujii S. Matsumoto S. Tajiri Y. Kikuchi A. Kiyoshima T. (2021). YAP signaling induces PIEZO1 to promote oral squamous cell carcinoma cell proliferation. J. Pathol. 253 (1), 80–93. 10.1002/path.5553 32985688

[B21] He J. Fang B. Shan S. Xie Y. Wang C. Zhang Y. (2021). Mechanical stretch promotes hypertrophic scar formation through mechanically activated cation channel Piezo1. Cell Death Dis. 12 (3), 226. 10.1038/s41419-021-03481-6 33649312 PMC7921104

[B22] He J. Fang B. Shan S. Li Q. (2023). Mechanical stiffness promotes skin fibrosis through Piezo1-mediated arginine and proline metabolism. Cell Death Discov. 9 (1), 354. 10.1038/s41420-023-01656-y 37752116 PMC10522626

[B23] He J. Cheng X. Fang B. Shan S. Li Q. (2024). Mechanical stiffness promotes skin fibrosis *via* Piezo1-Wnt2/Wnt11-CCL24 positive feedback loop. Cell Death Dis. 15 (1), 84. 10.1038/s41419-024-06466-3 38267432 PMC10808102

[B24] Holt J. R. Zeng W. Z. Evans E. L. Woo S. H. Ma S. Abuwarda H. (2021). Spatiotemporal dynamics of PIEZO1 localization controls keratinocyte migration during wound healing. Elife 10, e65415. 10.7554/eLife.65415 34569935 PMC8577841

[B25] Holt Y. Han Y. Kang J. Yu S. L. Park S. R. (2025). Piezo1 selectively enhances TGF-β1-induced IgA class switching by B cells. Cell Mol. Life Sci. 82 (1), 243. 10.1007/s00018-025-05789-4 40537609 PMC12179034

[B26] JiangZ. XuY. WangY. DongZ. HuW. SuJ. (2025). Matrix stiffness drives squamous cell carcinoma progression *via* a Piezo1-mediated mechanotransduction feedback loop. J. Adv. Res. 1232 (25). 10.1016/j.jare.2025.10.041 41151627

[B27] Jin C. Su S. Yu S. Zhang Y. Chen K. Xiang M. (2024). Essential roles of PIEZO1 in mammalian cardiovascular system: from development to diseases. Cells 13 (17), 1422. 10.3390/cells13171422 39272994 PMC11394449

[B28] Klingberg F. Chow M. L. Koehler A. Boo S. Buscemi L. Quinn T. M. (2014). Prestress in the extracellular matrix sensitizes latent TGF-β1 for activation. J. CELL Biol. 207 (2), 283–297. 10.1083/jcb.201402006 25332161 PMC4210443

[B29] Lai A. Thurgood P. Cox C. D. Chheang C. Peter K. Jaworowski A. (2022). Piezo1 response to shear stress is controlled by the components of the extracellular matrix. ACS Appl. Mater Interfaces 14 (36), 40559–40568. 10.1021/acsami.2c09169 36047858

[B30] Laschke M. W. Menger M. D. (2022). Microvascular fragments in microcirculation research and regenerative medicine. Tissue Eng. Part B Rev. 28 (5), 1109–1120. 10.1089/ten.TEB.2021.0160 34731017

[B31] Li M. Zhang X. Wang M. Wang Y. Qian J. Xing X. (2022). Activation of Piezo1 contributes to matrix stiffness-induced angiogenesis in hepatocellular carcinoma. Cancer Commun. (Lond) 42 (11), 1162–1184. 10.1002/cac2.12364 36181398 PMC9648387

[B32] Liao C. Wang P. Zeng Q. Yan G. Gao J. Liu J. (2025). Piezo1-Mediated calcium flux transfers mechanosignal to yes-associated protein to stimulate matrix production in keloid. J. Invest Dermatol 145 (25), 00415. 10.1016/j.jid.2025.03.039 40254148

[B33] Lin J. Wang S. Li Z. Li J. Wang X. Wan X. (2025). Da-yuan-yin decoction alleviates bleomycin-induced pulmonary injury by inhibiting epithelial-mesenchymal transition *via* E-cadherin/β-catenin complex restoration. J. Ethnopharmacol. 351, 120148. 10.1016/j.jep.2025.120148 40523450

[B34] Liu S. Xu X. Fang Z. Ning Y. Deng B. Pan X. (2021). Piezo1 impairs hepatocellular tumor growth *via* deregulation of the MAPK-Mediated YAP signaling pathway. Cell Calcium 95, 102367. 10.1016/j.ceca.2021.102367 33610907

[B35] Lüchtefeld I. Pivkin I. V. Gardini L. Zare-Eelanjegh E. Gäbelein C. Ihle S. J. (2024). Dissecting cell membrane tension dynamics and its effect on Piezo1-mediated cellular mechanosensitivity using force-controlled nanopipettes. Nat. Methods 21 (6), 1063–1073. 10.1038/s41592-024-02277-8 38802520 PMC11166569

[B36] Lv W. Yang F. Ge Z. Xin L. Zhang L. Zhai Y. (2024). Aberrant overexpression of myosin 1b in glioblastoma promotes angiogenesis *via* VEGF-myc-myosin 1b-Piezo1 axis. J. Biol. Chem. 300 (11), 107807. 10.1016/j.jbc.2024.107807 39307302 PMC11532902

[B37] Malko P. Jia X. Wood I. Jiang L. H. (2023). Piezo1 channel-mediated ca signaling inhibits lipopolysaccharide-induced activation of the NF-κB inflammatory signaling pathway and generation of TNF-α and IL-6 in microglial cells. Glia 71 (4), 848–865. 10.1002/glia.24311 36447422

[B38] Mei F. Guo Y. Wang Y. Zhou Y. Heng B. C. Xie M. (2024). Matrix stiffness regulates macrophage polarisation *via* the Piezo1-YAP signalling axis. Cell Prolif. 57 (8), e13640. 10.1111/cpr.13640 38556840 PMC11294424

[B39] Mills A. Aissaoui N. Maurel D. Elezgaray J. Morvan F. Vasseur J. J. (2022). A modular spring-loaded actuator for mechanical activation of membrane proteins. Nat. Commun. 13 (1), 3182. 10.1038/s41467-022-30745-2 35902570 PMC9334261

[B40] Mulhall E. M. Gharpure A. Lee R. M. Dubin A. E. Aaron J. S. Marshall K. L. (2023). Direct observation of the conformational states of PIEZO1. Nature 620 (7976), 1117–1125. 10.1038/s41586-023-06427-4 37587339 PMC10468401

[B41] Nam S. M. Kim Y. B. (2018). The effects of platelet-rich plasma on hypertrophic scars fibroblasts. Int. Wound J. 15 (4), 547–554. 10.1111/iwj.12896 29781178 PMC7949778

[B42] Papavassiliou K. A. Sofianidi A. A. Spiliopoulos F. G. Gogou V. A. Gargalionis A. N. Papavassiliou A. G. (2024). YAP/TAZ signaling in the pathobiology of pulmonary fibrosis. Cells 13 (18), 1519. 10.3390/cells13181519 39329703 PMC11430237

[B43] Pauty J. Nakano S. Usuba R. Nakajima T. Johmura Y. Omori S. (2021). A 3D tissue model-on-a-chip for studying the effects of human senescent fibroblasts on blood vessels. Biomater. Sci. 9 (1), 199–211. 10.1039/d0bm01297a 33174545

[B44] Peng F. Sun M. Jing X. Chen F. Cao T. Li Z. (2025). Piezo1 promotes intervertebral disc degeneration through the Ca^2+^/F-actin/Yap signaling axis. Mol. Med. 31 (1), 90. 10.1186/s10020-025-01147-z 40057686 PMC11889814

[B45] Rashidi N. Harasymowicz N. S. Savadipour A. Steward N. Tang R. Oswald S. (2025). PIEZO1-mediated mechanotransduction regulates collagen synthesis on nanostructured 2D and 3D models of fibrosis. Acta Biomater. 193, 242–254. Epub 2024. 10.1016/j.actbio.2024.12.034 39675497 PMC13276715

[B46] Rennekampff H. O. Tenenhaus M. Rennekampff I. Alharbi Z. (2024). Roles of mechanosensitive channel Piezo1 in wound healing and scar formation. Life (Basel) 14 (3), 377. 10.3390/life14030377 38541702 PMC10971801

[B47] Ridone P. Vassalli M. Martinac B. (2019). Piezo1 mechanosensitive channels: what are they and why are they important. Biophys. Rev. 11 (5), 795–805. 10.1007/s12551-019-00584-5 31494839 PMC6815293

[B48] Scott K. E. Fraley S. I. Rangamani P. (2021). A spatial model of YAP/TAZ signaling reveals how stiffness, dimensionality, and shape contribute to emergent outcomes. Proc. Natl. Acad. Sci. U. S. A. 118 (20), e2021571118. 10.1073/pnas.2021571118 33990464 PMC8157940

[B49] Shioka I. Morita R. Yagasaki R. Wuergezhen D. Yamashita T. Fujiwara H. (2024). *Ex vivo* SIM-AFM measurements reveal the spatial correlation of stiffness and molecular distributions in 3D living tissue. Acta Biomater. 189, 351–365. 10.1016/j.actbio.2024.09.023 39379233

[B50] Smith K. A. Chuntharpursat-Bon E. Povstyan O. V. Debant M. Kinsella J. A. Revill C. (2025). Regulation of PIEZO1 channel force sensitivity by interblade handshaking. Sci. Adv. 11 (24), eadt7046. 10.1126/sciadv.adt7046 40512861 PMC12164982

[B51] Sun W. Gao X. Lei H. Wang W. Cao Y. (2022). Biophysical approaches for applying and measuring biological forces. Adv. Sci. (Weinh) 9 (5), e2105254. 10.1002/advs.202105254 34923777 PMC8844594

[B52] Swain S. M. Romac J. M. Vigna S. R. Liddle R. A. (2022). Piezo1-mediated stellate cell activation causes pressure-induced pancreatic fibrosis in mice. JCI Insight 7 (8), e158288. 10.1172/jci.insight.158288 35451372 PMC9089793

[B53] Tu M. Lu C. Jia H. Chen S. Wang Y. Li J. (2024). SULF1 expression is increased and promotes fibrosis through the TGF-β1/SMAD pathway in idiopathic pulmonary fibrosis. J. Transl. Med. 22 (1), 885. 10.1186/s12967-024-05698-3 39354547 PMC11446151

[B54] Wang Y. Wang J. Zhang J. Wang Y. Wang Y. Kang H. (2024). Stiffness sensing *via* Piezo1 enhances macrophage efferocytosis and promotes the resolution of liver fibrosis. Sci. Adv. 10 (23), eadj3289. 10.1126/sciadv.adj3289 38838160 PMC11152137

[B55] Wu W. S. Chen L. R. Chen K. H. (2025). Platelet-rich plasma (PRP): molecular mechanisms, actions and clinical applications in human body. Int. J. Mol. Sci. 26 (21), 10804. PMID: 41226837. 10.3390/ijms262110804 41226837 PMC12608683

[B56] Xu L. Li T. Cao Y. He Y. Shao Z. Liu S. (2025). PIEZO1 mediates periostin+ myofibroblast activation and pulmonary fibrosis in mice. J. Clin. Invest 135 (11), e184158. 10.1172/JCI184158 40454481 PMC12126248

[B57] Xue Y. Winnicki E. Zhang Z. Lopez I. Wang S. Kirby C. (2025). The mechanotransducer Piezo1 coordinates metabolism and inflammation to promote skin growth. Nat. Commun. 16 (1), 6876. 10.1038/s41467-025-62270-3 40715139 PMC12297709

[B58] Yan W. Maimaitimin M. Wu Y. Fan Y. Ren S. Zhao F. (2023). Meniscal fibrocartilage regeneration inspired by meniscal maturational and regenerative process. Sci. Adv. 9 (45), eadg8138. 10.1126/sciadv.adg8138 37939174 PMC10631723

[B59] Yang F. Zhang W. (2025). Autologous fat granules combined with PRP treat hypertrophic scars through TGF-β/Smad signaling pathway. Aesthet. Plast. Surg. 49 (19), 5604–5610. 10.1007/s00266-025-05136-9 40826296

[B60] Yang S. A. Carpenter C. L. Abrams C. S. (2004). Rho and rho-kinase mediate thrombin-induced phosphatidylinositol 4-phosphate 5-kinase trafficking in platelets. J. Biol. Chem. 279 (40), 42331–42336. 10.1074/jbc.M404335200 15277528

[B61] Yang P. Liu H. Wang S. Xiao X. Jiang L. Le S. (2025). PIEZO1 attenuates Marfan syndrome aneurysm development through TGF-β signaling pathway inhibition *via* TGFBR2. Eur. Heart J. 46 (10), 958–974. 10.1093/eurheartj/ehae786 39585648

[B62] Yu Y. Liu P. F. Zhan M. W. Chen Y. Q. Lai Y. Q. Wang L. (2025). Lysyl oxidase promotes seminal vesicle atrophy and fibrosis in type-2 diabetes mellitus through TGF-β1 signaling. J. Transl. Med. 23 (1), 854. 10.1186/s12967-025-06590-4 40739261 PMC12312368

[B63] Zare S. Zeinali R. Nouri M. Dehghani A. Zare S. Khatib S. (2025). Comparison combination of autologous fibroblast cells plus platelet rich plasma (PRP) with PRP alone in treatment of atrophic acne scars, a split-face pilot study with biometric assessment. J. Cosmet. Dermatol 24 (9), e70413. 10.1111/jocd.70413 40853048 PMC12376645

[B64] Zhang W. Jiang H. Kong Y. (2020). Exosomes derived from platelet-rich plasma activate YAP and promote the fibrogenic activity of müller cells *via* the PI3K/Akt pathway. Exp. Eye Res. 193, 107973. 10.1016/j.exer.2020.107973 32059976

[B65] Zhao X. Kong Y. Liang B. Xu J. Lin Y. Zhou N. (2022). Mechanosensitive Piezo1 channels mediate renal fibrosis. JCI Insight 7 (7), e152330. 10.1172/jci.insight.152330 35230979 PMC9057604

[B66] Zhou J. Liu Y. Liu X. Wan J. Zuo S. Pan T. (2023). Hyaluronic acid-based dual network hydrogel with sustained release of platelet-rich plasma as a diabetic wound dressing. Carbohydr. Polym. 314, 120924. 10.1016/j.carbpol.2023.120924 37173024

[B67] Zhu Y. Xiao F. Wang Y. Wang Y. Li J. Zhong D. (2025). NINJ1 regulates plasma membrane fragility under mechanical strain. Nature 644 (8078), 1088–1096. 10.1038/s41586-025-09222-5 40490006 PMC12210241

[B68] Zhuang Y. Yin T. Li J. Zang Y. Li X. (2024). An allysine-conjugatable probe for fluorogenically imaging fibrosis. Anal. Chem. 96 (22), 9034–9042. 10.1021/acs.analchem.4c00404 38773734

